# Update on Diagnostic Performance of PET/MRI in Gynecological Malignancies: A Systematic Review and Meta-Analysis

**DOI:** 10.5334/jbsr.1981

**Published:** 2020-01-23

**Authors:** Mayur Virarkar, Catherine Devine, Roland Bassett, Sanaz Javadi, Silvana De Castro Faria, Priya Bhosale

**Affiliations:** 1 Department of Diagnostic Radiology, The University of Texas MD Anderson Cancer Center, Houston, Texas, US; 2 Department of Biostatistics, The University of Texas MD Anderson Cancer Center, Houston, Texas, US

**Keywords:** PET/MRI, gynecological, meta-analysis

## Abstract

**Objective:**

The aim of this study was to assess the diagnostic performance of ^18^F-fluorodeoxyglucose (FDG) positron emission tomography/magnetic resonance imaging (PET/MRI) for gynecological cancers of the pelvis based on a systematic review and meta-analysis of published data.

**Patients and Methods:**

A systematic literature search for original diagnostic studies was performed using PubMed/MEDLINE, the Cochrane Library, Embase and Web of Science. The methodological quality of each study was evaluated using the Quality Assessment of Diagnostic Accuracy Studies-2 tool. Data necessary for entry in 2 × 2 contingency tables were obtained, and patients, study, and imaging characteristics were extracted from the selected articles. Statistical analysis included data pooling, heterogeneity testing, sensitivity analyses, forest plotting, and summary receiver operating characteristic curve construction.

**Result:**

Twelve studies met our predefined inclusion criteria and were included in this study. Patient-based analysis, the pooled sensitivity rate, specificity rate, diagnostic odds ratio, and area under the receiver operating characteristic curve for ^18^F-FDG PET/MRI in diagnosis of gynecological malignancies were 74.2% (95% confidence interval, 66.2–80.8%), 89.8% (95% CI, 82.2–94.3%), 26 (95% CI, 10–67), and 0.834, respectively. On lesion-based analysis, the pooled sensitivity rate, specificity rate, diagnostic odds ratio, and area under the curve were 87.5% (95% CI, 75.8–94.0%), 88.2% (95% CI, 84.2–91.3%), 50 (95% CI, 23–111), and 0.922, respectively.

**Conclusions:**

Our meta-analysis demonstrated that ^18^F-FDG PET/MRI is a promising diagnostic method for primary tumors, nodal staging, and recurrence in patients with gynecological malignancies of the pelvis.

Gynecological malignancies are among the leading causes of cancer deaths in women [1]. Accurate staging and assessment for recurrence are of paramount importance in their management and improving survival. Imaging is indispensable in evaluating these malignancies. Positron emission tomography/magnetic resonance imaging (PET/MRI) permits integrated high-quality imaging of primary tumors and metastases. PET/MRI has become a powerful technology, providing metabolic information via PET and high-resolution anatomic information and functional imaging properties via MRI (Figure 1). Several studies have demonstrated the diagnostic potential of this hybrid imaging modality in the management of gynecological cancers.

**Figure 1:**
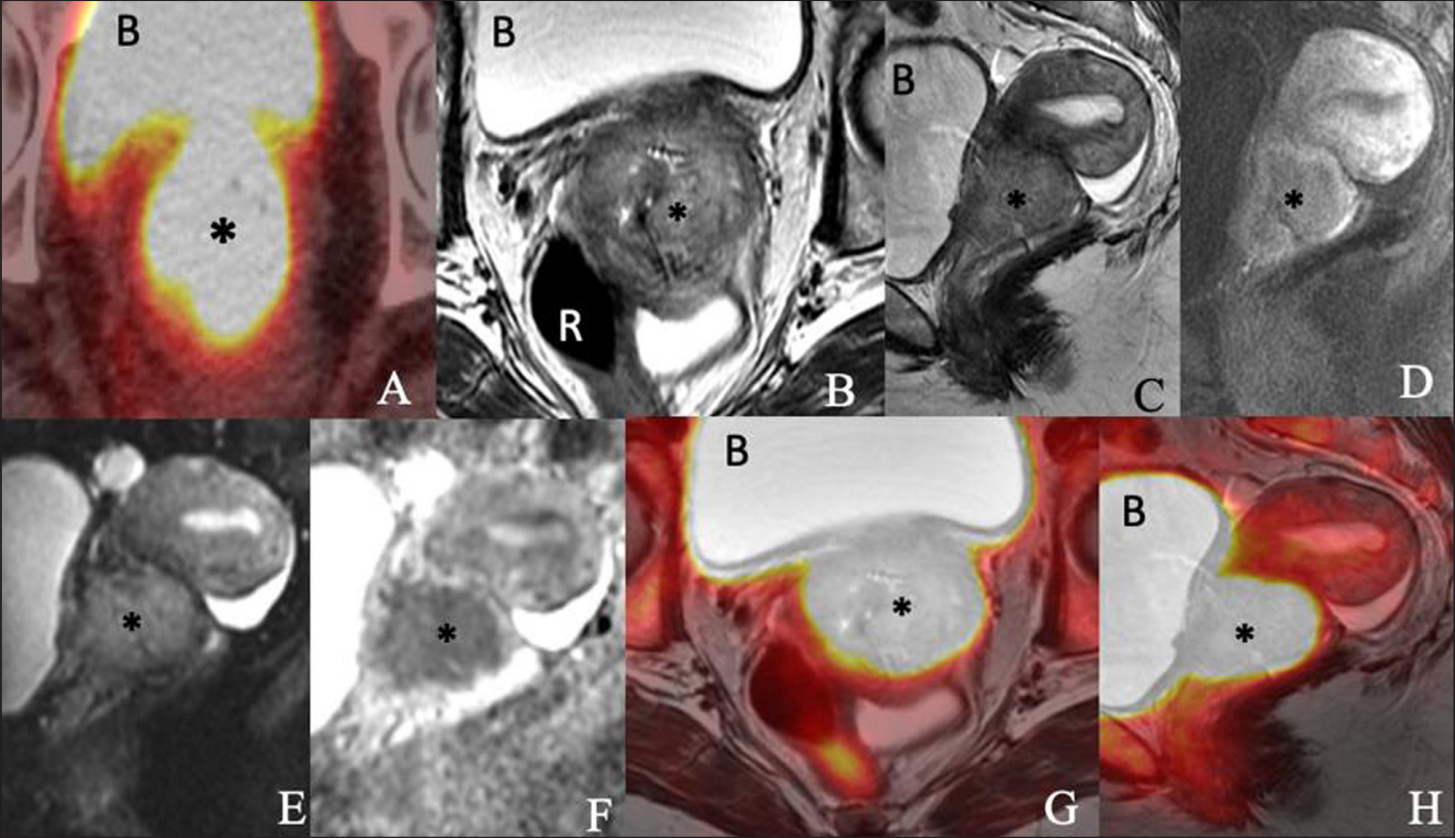
Images of a 76-year-old woman with newly diagnosed squamous cell carcinoma of the cervix consisting of an **(A)** axial PET/CT, **(B)** axial T2-weighted, **(C)** sagittal T2-weighted, **(D)** sagittal postcontrast T1-weighted, **(E)** sagittal diffusion-weighted **(F)** sagittal apparent diffusion coefficient map of MRI, **(G)** axial T2-weighted PET/MRI and **(H)** sagittal T2-weighted PET/MRI images show a 5.4 × 4.6 × 4.1-cm enhancing FDG-avid cervical mass (*) invading the parametrium and extending into the vaginal fornices and lower uterine segment. The mass exhibits restricted diffusion. The bladder (B) and rectum (R) appear to be uninvolved.

The aim of this systematic review and meta-analysis was to evaluate the diagnostic performance of ^18^F-fluorodeoxyglucose (FDG) PET/MRI for gynecological malignancies of the pelvis.

## Patients and Methods

### Database Search Strategy

**Figure 2:**
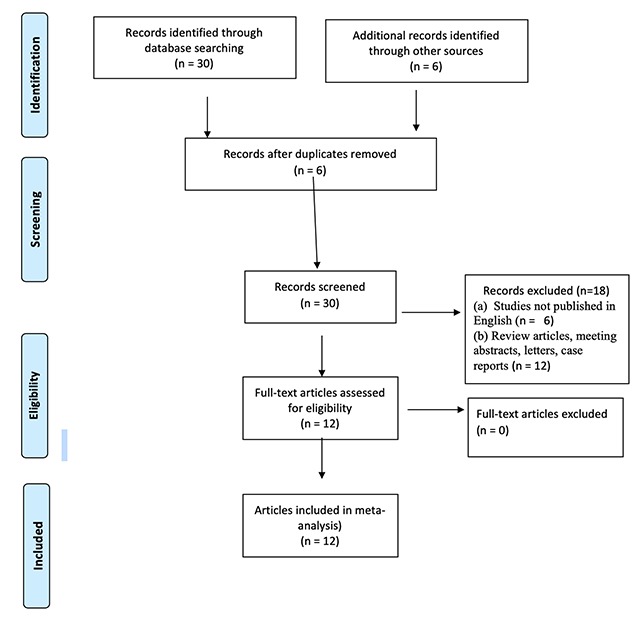
Preferred Reporting Items for Systematic Reviews and Meta-Analyses (PRISMA) flow diagram of the meta-analysis.

The Preferred Reporting Items for Systematic Reviews guidelines were used in reporting the results of this study [2]. All available literature in the PubMed/MEDLINE, Web of science, Embase, and Cochrane Library databases published through May 2019 were searched. The databases were comprehensively searched using the following “PET/MRI” or “PET/MR” or “PETMRI” or “PET-MR” or “positron emission tomography magnetic resonance imaging” or “positron emission tomography/magnetic resonance imaging” AND “cervical” or “cervix” or “endometrial” or “ovarian” or “vaginal” or “vulvar” or “gynecological” AND “cancer” or “carcinoma” or “neoplasm”. The reference lists in all of the retrieved studies were scrutinized for additional articles to supplement the search results (Figure 2).

### Inclusion Criteria

Studies were eligible for inclusion if 1) the diagnostic performance of PET/MRI for gynecological malignancies was clearly identified in the study; 2) the number of true-positive (TP), true-negative (TN), false-positive (FP), and false-negative (FN) results could be obtained from the article; and 3) the reference standard for malignancy was either histopathological analysis or imaging follow-up.

### Exclusion Criteria

The following types of articles were excluded: 1) articles not published in English and 2) review articles, meeting abstracts, letters, case reports, and articles without sufficient data to construct a 2 × 2 contingency table.

### Study Quality Assessment

The literature was searched and the quality of all eligible studies were assessed using the current Quality Assessment of Diagnostic Accuracy Studies-2 (QUADAS-2) tool [3]. This tool is composed of four major domains: patient selection, index test, reference standard, and flow and timing. These domains were then further assessed on the basis of the risk of bias, and their applicability was rated as high, low, or unclear. A co-author (P.B.) was consulted to assess the accuracy of the data.

### Data Extraction

The articles were reviewed to collect all key information (e.g., study design, country of patient recruitment, technical specifications). Any disagreements about data extraction were resolved by consensus among the co-authors. The numbers of TP, FP, TN, and FN results were obtained or derived from the studies. In some of the studies, lesions were assessed on a per-patient basis. Hence, the data was included in the patient-based data group and subsequent data analysis.

### Diagnostic Performance Analysis

Our primary diagnostic performance analysis of PET/MRI included only studies in which PET/MRI was performed in the same population in an attempt to reduce the clinical (i.e., pretest probability of malignancy) and methodological heterogeneity.

### Statistical Analysis

Statistical Analyses were performed separately for the lesion- and patient-level data. Analyses were performed for the study group to estimate the sensitivity, specificity, diagnostic odds ratio (DOR) and area under the receiver operating characteristic curve (AUC). Summary tables and forest plots were used to summarize the results. The pooled sensitivity and specificity rates and DORs were estimated using a random effects model with the approach described by DerSimonian and Laird [4]. Summary receiver operating characteristic (SROC) curves were used to summarize the relationship between sensitivity and specificity, DOR and the AUC. The heterogeneity of our study was assessed using the Cochran Q test and Higgins I^2^. Furthermore, Deeks’ funnel plot was used to assess publication bias. All statistical analyses were performed using the R computing language (version 3.6.0). All statistical tests used a significance level of 5%. No adjustments for multiple testing were made.

## Results

### Study Selection and Description

The initial search yielded 36 articles on studies involving PET/MRI in patients with gynecological malignancies 12 of which were eligible for inclusion in our analyses. The characteristics of these studies are summarized in Table 1 [5,6,7,8,9,10,11,12,13,14,15,16].

**Table 1:** Characteristics of the 12 Studies of PET/MRI Performed in the Same Patient Population.

Reference	Year	Country	PET/MRI or Fusion	Study Type	Number of Subjects	Primary Finding	Objective

Kim et al. [5]	2009	Republic of Korea	Signa 1.5T system (GE Healthcare, Milwaukee, WI) Image fusion: Advantage Workstation (version 4.3; GE Healthcare)	Retrospective	79	Cervical cancer (n = 79)	Staging
Fiaschetti et al. [6]	2011	Italy	3T permanent magnet (Achieva; Philips, Best, Netherlands) Image fusion: Advantage MR-PET Fusion on Advantage Workstation (version 4.4; GE Healthcare)	Prospective	24	Ovarian lesions: malignant (n = 19), benign (n = 5)	Staging
Kitajima et al. [7]	2013	Japan	1.5T MR scanner (Signa EchoSpeed Plus Excite 1.5T; GE Healthcare) Image fusion: Advantage Workstation (version 4.5; GE Healthcare)	Retrospective	30	Endometrial cancer (n = 35)	Staging
Kitajima et al. [9]	2014	Japan	1.5T MR scanner (Achieva; Philips) Image fusion: Advantage Workstation (version 4.5; GE Healthcare)	Retrospective	35	Cervical cancer (n = 35)	Staging
Kitajima et al. [10]	2014	Japan	1.5T MR scanner (Achieva; Philips) Image fusion: Advantage Workstation (version 4.5; GE Healthcare)	Retrospective	30	Locally recurrent disease (n = 16), pelvic lymph node metastases (n = 8), bone metastases (n = 3), peritoneal dissemination (n = 5)	Recurrence and metastatic disease
Grueneisen et al. [8]	2014	Germany	3T PET/MRI Biograph scanner (Siemens Healthineers, Erlangen, Germany)	Prospective	48	Primary cancer (n = 27), recurrence (n = 21)	Staging and recurrence
Queiroz et al. [13]	2015	Switzerland	3T Discovery MR750w (GE Healthcare) Image fusion: Advantage Workstation (version 4.5; GE Healthcare)	Prospective	26	Ovarian (n = 12), cervical (n = 7), endometrial (n = 4), vulvar (n = 1), and primary peritoneal (n = 1) cancer and uterine metastasis (n = 1)	Staging
Grueneisen et al. [12]	2015	Germany	3T PET/MRI Biograph scanner (Siemens Healthineers)	Prospective	24	Ovarian (n = 13), cervical (n = 7), and endometrial (n = 4) cancer	Recurrence
Grueneisen et al. [11]	2015	Germany	3T PET/MRI Biograph scanner (Siemens Healthineers)	Prospective	27	Primary cervical cancer (n = 27)	Staging
Stecco et al. [14]	2016	Italy	1.5T MRI scanner (Achieva Intera; Philips) Image fusion: Leonardo multimodality workstation (Siemens Healthineers)	Retrospective	27	Cervical (n = 14) and endometrial (n = 13) cancers	Staging
Kirchner et al. [15]	2017	Germany	3T PET/MRI scanner (Biograph mMR; Siemens Healthineers)	Prospective	43	Ovarian (n = 23), cervical (n = 12), endometrial (n = 4), vulvar (n = 3), and vaginal (n = 1) cancers	Recurrence
Mongula et al. [16]	2018	Netherlands	3T PET/MRI scanner (Biograph mMR; Siemens Healthineers)	Prospective	10	Cervical cancer (n = 10)	Response assessment after radiation therapy

### QUADAS-2 Assessment

**Figure 3:**
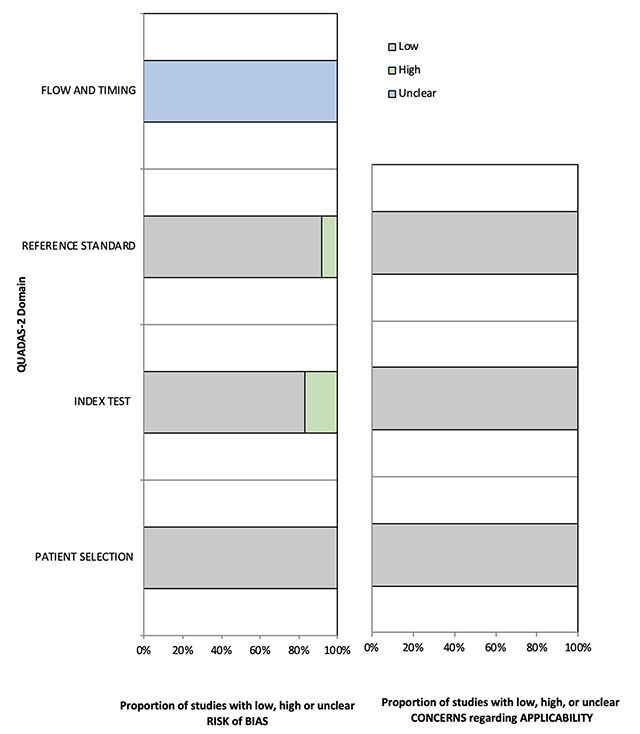
Methodological quality of all eligible studies according to QUADAS-2.

The distribution of the QUADAS-2 scores for the methodological quality (i.e., risk of bias and concerns regarding applicability) of the 12 studies is presented in Table 2 and Figure 3. Most of the assessed studies have a low risk of bias and few concerns regarding applicability. Regarding patient selection, the studies had a low risk of bias because of avoidance of case-control design and inappropriate exclusion criteria. Concerning index testing, all but two studies had a low risk of bias because of blinding to the reference test, except two studies [5,11]. In reference testing, the studies had a low risk of bias. All of the researchers in these studies used reference standards. Investigators used histological confirmation and imaging follow-up as the reference standards, except one study, which used only imaging follow-up as the reference standard [16]. Regarding flow and timing, all of the studies had an unclear risk of bias because the researchers did not provide an appropriate interval between the index test and reference standard. We did not exclude any studies from the analysis on the basis of the methodological quality assessment.

**Table 2:** Tabular presentation of QUADAS-2 results of the selected articles.

Study	Risk of Bias				Applicability Concerns		

	Patient Selection	Index Test	Reference Standard	Flow and Timing	Patient Selection	Index Test	Reference Standard

Kim et al. [5]	☺	☹	☺	**?**	☺	☺	☺
Fiaschetti et al. [6]	☺	☺	☺	**?**	☺	☺	☺
Kitajima et al. [7]	☺	☺	☺^†^	**?**	☺	☺	☺
Kitajima et al. [9]	☺	☺	☺^†^	**?**	☺	☺	☺
Kitajima et al. [10]	☺	☺	☺^†^	**?**	☺	☺	☺
Queiroz et al. [13]	☺	☺	☺^†^	**?**	☺	☺	☺
Grueneisen et al. [12]	☺	☺	☺^†^	**?**	☺	☺	☺
Grueneisen et al. [8]	☺	☺	☺	**?**	☺	☺	☺
Grueneisen et al. [11]	☺	☹	☺	**?**	☺	☺	☺
Stecco et al. [14]	☺	☺	☺	**?**	☺	☺	☺
Kirchner et al. [15]	☺	☺	☺^†^	**?**	☺	☺	☺
Mongula et al. [16]	☺	☺	☹	**?**	☺	☺	☺

^†^ Reference standards included histopathology and imaging follow up.

### Diagnostic Performance of PET/MRI

Some of the 12 articles we analyzed had patient-level data, some had lesion-level data, and some had both. The articles provided data on 20 patient-level groups and 10 lesion-level groups in the form of TP, TN, FP, and FN. In one of the lesion-level groups, the TN, FP, and FN were all zero [13]. Thus, we could not use summary statistics or conduct statistical analyses. In another study the TN and FP were both zero in the lesion-level group [6]. We excluded these two groups from all analyses, leaving a total of eight lesion-level groups. In addition, in one of the patient-level groups, the TN and FP were both zero [15]. We also excluded this group from our analyses, leaving a total of 19 patient-level groups. In cases in which at least a TP, TN, FP, or FN was zero, we added a correction factor of 0.5 to all cells to make all calculations finite.

### Patient-Level Analysis

**Figure 4:**
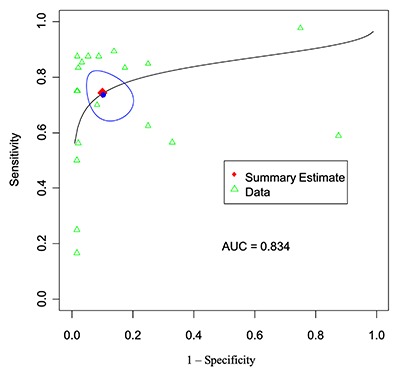
Patient-level analysis: SROC curve for PET-MRI.

As described above, we included a total of 19 patient-level groups from 8 articles. For ^18^F-FDG PET/MRI in diagnosis of gynecological malignancies, the pooled sensitivity rate was 74.2% (95% confidence interval (6), 66.2–80.8%), and the pooled specificity rate was 89.8% (95% CI, 82.2–94.3%). Also, the pooled DOR was 26 (95% CI, 10–67), and the AUC was 0.834 (Table 3). We also performed two assessments of the heterogeneity of the studies for DOR, The Cochran Q was 14.2 (*P* = 0.72), indicating no evidence of heterogeneity. In addition, the Higgins I^2^ was 0, also demonstrating no evidence of heterogeneity. An SROC curve for the patient-level PET/MRI data is shown in Figure 4.

**Table 3:** Diagnostic Performance of PET/MRI in Imaging of Gynecological Malignancies (Patient-Based Analysis).

Parameter	PET/MRI	95% CI

No. of TP results	179	–
No. of TN results	346	–
No. of FP results	30	–
No. of FN results	45	–
Sensitivity (%)	74.2	66.2–80.8
Specificity (%)	89.8	82.2–94.3
DOR	26	10–67
AUC	0.834	–

### Lesion-Level Analysis

**Figure 5:**
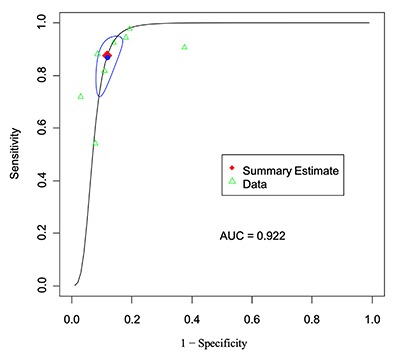
Lesion-level analysis: SROC curve for PET-MRI.

Also as described above, we included a total of eight lesion-level groups from six articles. The pooled sensitivity rate for PET/MRI was 87.5% (95% CI, 75.8–94.0%), and the pooled specificity rate was 88.2% (95% CI, 84.2–91.3%). Furthermore, the pooled DOR for PET/MRI was 50 (95% CI, 23–111), and the AUC was 0.922 (Table 4). We performed two assessments of the heterogeneity of the studies the lesion-level data. The Cochran Q was 4.5 (*P* = 0.72), and the Higgins I^2^ was 0, both indicating no evidence of heterogeneity. An SROC curve for the lesion-level PET/MRI data is shown in Figure 5.

**Table 4:** Diagnostic Performance of PET/MRI in Imaging of Gynecological Malignancies (Lesion-Based Analysis).

Parameter	PET/MRI	95% CI

No. of TP results	496	–
No. of TN results	730	–
No. of FP results	70	–
No. of FN results	67	–
Sensitivity (%)	87.5	75.8–94.0
Specificity (%)	88.2	84.2–91.3
DOR	50	23–111
AUC	0.922	–

Deeks’ funnel plot regression revealed no statistical evidence of publication bias in PET/MRI in lesion level data. The numbers of PET/MRI studies were, however, insufficient to allow for assessment of reporting bias.

## Discussion

Our meta-analysis demonstrated a pooled sensitivity rate, specificity rate, DOR, and AUC for ^18^F-FDG PET/MRI in diagnosis of gynecological malignancies of 74.2% (95% CI, 66.2–80.8%), 89.8% (95% CI, 82.2–94.3%), 26 (95% CI, 10–67), and 0.834, respectively, in patient-based assessment. In lesion-based assessment, these numbers were 87.5% (95% CI, 75.8–94.0%), 88.2% (95% CI, 84.2–91.3%), 50 (95% CI, 23–111), and 0.922, respectively (Tables 3 and 4).

Kim and colleagues conducted the earliest study of the fusion of MRI and PET and comparing it with PET/CT for detection of metastatic lymphadenopathy in patients with cervical malignancies (n = 27) [5]. The patients underwent MRI and PET/CT followed by surgical lymphadenectomy. They initially underwent MRI and PET/CT followed by analysis of fused PET/MRI images. Histopathological evaluation of the lymph nodes was the diagnostic reference standard. They reported identification of more metastatic lymph nodes (n = 6) with fused PET/MRI than with PET/CT. Both had a comparable sensitivity (54.2% vs. 44.1%) and specificity (92.7% vs. 93.9%), but better diagnostic performance of PET/MRI (*P* = 0.0259). They also found a significant difference in the AUC in detection of viable tumors between PET/CT and fused PET/MRI (*P* = 0.045). Some potential limitations of this study included nonblinding of readers to the MRI findings during interpretation of PET/CT images, suboptimal evaluation of the tumor on noncontract PET/CT, and verification bias of the surgeon due to due to knowledge of preoperative PET and MRI.

Kitajima and colleagues conducted three studies of the usefulness of PET/MRI in diagnosing gynecological cancers and its diagnostic performance in comparison with that of PET/CT. In their initial study for staging of endometrial cancer (n = 30 patients), they performed preoperative contrast PET/CT, MRI, and then retrospective PET/MRI [7]. They used histopathological analysis and imaging follow-up as the reference standards. They reported a higher primary tumor detection rate (96.7% vs. 93.0%) and significantly better results in identifying the T stage (80% vs. 60%; *P* = 0.041) with PET/MRI than with PET/CT. The reported identical patient-based sensitivity (100%) and specificity (96.3%) rates and accuracy (96.7%) in detection of pelvic node metastases. In another study by this group on the use of PET/MRI and PET/CT for diagnosis of recurrent gynecological malignancies, the authors reported higher patient-based sensitivity of PET/MRI in detecting local recurrence (87.5%), pelvic lymph node metastasis (87.5%), bone metastasis (100%), and peritoneal lesions (80%) [10]. The sensitivity in detecting local recurrence was significantly better for PET/MRI than for PET/CT (87.5% vs. 62.5%; *P* = 0.041). In the third study by Kitajima’s group, which was on staging of uterine and cervical cancers (n = 35 patients), PET/MRI was significantly more accurate in staging than PET/CT (83.0% vs. 53.3%; *P* = 0.0077), with sensitivity and specificity rates and accuracy in detection/diagnosis of nodal metastasis of 92.3%, 88.2%, and 90.0%, respectively [9]. However, the limitations of this study included failure to perform whole-body MRI, resulting in inability to evaluate distant metastases, misregistration of fusion images, and unavailability of histopathological confirmation in all patients and lesions.

Similarly, Grueneisen and colleagues conducted three studies evaluating the diagnostic performance of PET/MRI for female pelvic malignancies. In the earliest study, they assessed the value of addition of diffusion-weighted imaging to PET/MRI for diagnosis of primary and recurrent pelvic malignancies (n = 48 patients) [8]. They reported sensitivity (92.9%, 94.9%), specificity (87.5%, 83.3%), positive predictive value (PPV; 96.8%, 95.9%), negative predictive value (NPV; 75%, 80%), and diagnostic accuracy (91.8%, 92.6%) for detection of malignant lesions with and without diffusion-weighted imaging to the PET/MRI, respectively. The inclusion of diffusion-weighted imaging in the PET/MRI had no added benefit. They used histopathological analysis and imaging follow-up as the reference standards.

In another study, this group assessed the diagnostic value of PET/MRI in staging cervical cancer (n = 27 patients) [11]. PET/MRI identified all 27 primary tumors, with 85% accuracy (23/27) in determination of the T stage. Also, the sensitivity and specificity rates and accuracy for nodal detection (n = 11) were 91%, 94%, and 93%, respectively. The researchers used histopathological confirmation as the reference standard. In addition, the investigators found a significant association of functional parameters (standardized uptake value and apparent diffusion coefficient) with pathological grade and tumor size (*P* < 0.05). The authors also pointed out that the functional parameters correlation results were based on a small group of patients and presented as preliminary results, remaining to be confirmed in larger cohorts.

In the third study, Grueneisen’s group compared the diagnostic performance of PET/MRI and PET/CT for recurrence of pelvic malignancies (n = 24 patients) [12]. The two modalities identified similar numbers of tumor recurrences (20 out of 21). The lesion-based sensitivity and specificity rates, PPV, NPV, and diagnostic accuracy in the detection of malignant lesions were not significantly different for PET/CT and PET/MRI (82% vs. 85%, 91% vs. 87%, 97% vs. 96%, 58% vs. 63%, and 84% vs. 86%, respectively; *P* > 0.05). This study was potentially biased due to the influence of earlier performed PET/CT findings on interpretation of PET/MRI.

In another comparative study of PET/CT and fused PET/MRI for the detection of cervical and endometrial cancer lymph node metastases (n = 27 patients), investigators reported the similar sensitivity rate (87.5%), specificity rate (84.2%), diagnostic accuracy (85.1%), PPV (70%), and NPV (94.1%) for PET/MRI as for PET/CT on a per-patient basis [14]. On a per-node basis, PET/MRI had better sensitivity (89.0% vs. 70.2 %), specificity (91.6% vs. 90.5%), diagnostic accuracy (91.2% vs. 87.0%), PPV (68.7% vs. 60.4%), and NPV (97.6% vs. 93.6%) than did PET/CT but without a significant AUC (*P* = 0.0055). However, they obtained fused PET/MRI scans of only the pelvis rather than the whole body, and they performed histopathological confirmation for only a few patients (n = 8).

Queiroz et al. explored the benefits of PET/MRI in diagnosis of advanced gynecological tumors (n = 26 patients) [13]. They reported superiority of PET/MRI over PET/CT in tumor delineation in 12 of 17 patients. The tumor delineation parameters included parametrial/upper third of the vagina (n = 6); relation to surrounding structures like vessels and the bladder, rectum, and abdominal wall (n = 4); myometrial invasion (n = 3); and tumor characterization (n = 1). PET/MRI and PET/CT both accurately detected primary and recurrent tumors (n = 24) and regional (n = 11) and abdominal (n = 14) metastases. However, PET/MRI did not detect distant extra-abdominal metastases (n = 5). Also, they did not perform whole-body MRI and hence were unable to determine the M stage in the patients. The researchers used histopathological confirmation and imaging follow-up as the reference standards.

The longer scanning time for PET/MRI examination is often considered as its drawback. Hence, Kirchner et al. used ultrafast PET/MRI in staging recurrent pelvic malignancies (n = 43) and compared the performance with that of PET/CT [15]. The ultrafast PET/MRI parameters consisted of coronal T1-weighted volumetric interpolated breath-hold examination (VIBE), Dixon chemical shift, axial T2-weighted half-Fourier acquisition single-shot turbospin echo (HASTE), and axial T1-weighted VIBE post contrast sequences. Both PET/MRI and PET/CT had a similar scan durations (18.5 minutes vs. 18.2 minutes). PET/CT enabled slightly more correct identification of patients with recurrent cancer (37/38) than did PET/MRI (36/38). In a lesion-based analysis, the sensitivity rate, specificity rate, PPV, NPV, and diagnostic accuracy were 97%, 83%, 93%, 94%, and 92%, respectively, for PET/CT and 98%, 83%, 94%, 94%, and 94%, respectively, for PET/MRI. PET/CT missed three malignant lesions and PET/MRI falsely identified only two malignant lesions as benign. Histopathological confirmation and imaging follow-up were used as the reference standards.

In a similar study designed to assess the use of PET/MRI for characterization of ovarian lesions (n = 24), PET/CT had a sensitivity rate, specificity rate, PPV, and NPV of 74%, 80%, 93%, and 44%, respectively, whereas these values for PET/MRI were 94%, 100%, 100%, and 83%, respectively [6]. PET/CT detected 14 of 19 malignant lesions (74%), whereas PET/MRI detected 18 of 19 malignant lesions (95%). The mean SUVmax value for pathological findings of PET/CT was 5.0 ± 2.3, and that for PET/MRI was 4.2 ± 2.0. The mean size of the lesions diagnosed as malignant using PET/MRI was 45 ± 29 mm. The researchers used histopathological confirmation as the reference standard. This study was the first to compare the SUVmax and tumor size for PET/MRI, which should be investigated in future studies.

PET/MRI has potential in the assessment of treatment response of gynecological malignancies. Mongula et al. evaluated the response of International Federation of Gynecology and Obstetrics stage IIB2 or higher cervical carcinoma (n = 10 patients) to radiotherapy after 11 weeks of treatment [16]. Three patients had residual tumor after treatment. The utilization of PET/MRI increased the diagnostic confidence (80–90%) and resulted in a change of opinion on diagnosis (70%) and change in management plan of the patients (50%). PET/MRI also markedly increased the diagnostic accuracy for the radiologist, when PET/MRI was combined (AUC, 0.54 vs. 0.83). Histopathological confirmation was not available and imaging follow-up was used as the reference standard.

Our study had some limitations. For example, the studies included in the meta-analysis were highly heterogeneous. Also, some studies used PET/MRI for cancer staging, whereas others utilized it for detection of metastases and tumor recurrence. In addition, no publication bias was found, but the bias cannot be excluded entirely. The studies further lacked optimal standardized guidelines for PETMRI, scanning protocols and flow of timing. Finally, the numbers of included studies and patients were not large.

In conclusion, ^18^F-FDG PET/MRI is a promising imaging modality with high sensitivity and specificity and excellent diagnostic performance in assessment of pelvic gynecological malignancies. More randomized controlled trials with increasing numbers of patients are recommended to enhance its diagnostic performance.

## References

[1] Torre LA, Islami F, Siegel RL, Ward EM, Jemal A. Global Cancer in Women: Burden and Trends. Cancer Epidemiol Biomarkers Prev. 2017; 26(4): 444–57. DOI: 10.1158/1055-9965.EPI-16-0858 28223433

[2] Moher D, Liberati A, Tetzlaff J, Altman DG. Preferred reporting items for systematic reviews and meta-analyses: the PRISMA statement. International Journal of Surgery (London, England). 2010; 8(5): 336–41. DOI: 10.1016/j.ijsu.2010.02.007 20171303

[3] Whiting PF, Rutjes AW, Westwood ME, et al. QUADAS-2: A revised tool for the quality assessment of diagnostic accuracy studies. Annals of Internal Medicine. 2011; 155(8): 529–36. DOI: 10.7326/0003-4819-155-8-201110180-00009 22007046

[4] DerSimonian R, Laird N. Meta-analysis in clinical trials. Control Clin Trials. 1986; 7(3): 177–88. DOI: 10.1016/0197-2456(86)90046-2 3802833

[5] Kim SK, Choi HJ, Park SY, et al. Additional value of MR/PET fusion compared with PET/CT in the detection of lymph node metastases in cervical cancer patients. European Journal of Cancer (Oxford, England: 1990). 2009; 45(12): 2103–9. DOI: 10.1016/j.ejca.2009.04.006 19403303

[6] Fiaschetti V, Calabria F, Crusco S, et al. MR-PET fusion imaging in evaluating adnexal lesions: a preliminary study. La Radiologia medica. 2011; 116(8): 1288–302. DOI: 10.1007/s11547-011-0720-7 21892714

[7] Kitajima K, Suenaga Y, Ueno Y, et al. Value of fusion of PET and MRI for staging of endometrial cancer: comparison with ^18^F-FDG contrast-enhanced PET/CT and dynamic contrast-enhanced pelvic MRI. European Journal of Radiology. 2013; 82(10): 1672–6. DOI: 10.1016/j.ejrad.2013.05.005 23727380

[8] Grueneisen J, Schaarschmidt BM, Beiderwellen K, et al. Diagnostic value of diffusion-weighted imaging in simultaneous ^18^F-FDG PET/MR imaging for whole-body staging of women with pelvic malignancies. J Nucl Med. 2014; 55(12): 1930–5. DOI: 10.2967/jnumed.114.146886 25453042

[9] Kitajima K, Suenaga Y, Ueno Y, et al. Fusion of PET and MRI for staging of uterine cervical cancer: Comparison with contrast-enhanced ^18^F-FDG PET/CT and pelvic MRI. Clin Imaging. 2014; 38(4): 464–9. DOI: 10.1016/j.clinimag.2014.02.006 24642250

[10] Kitajima K, Suenaga Y, Ueno Y, et al. Value of fusion of PET and MRI in the detection of intra-pelvic recurrence of gynecological tumor: Comparison with ^18^F-FDG contrast-enhanced PET/CT and pelvic MRI. Ann Nucl Med. 2014; 28(1): 25–32. DOI: 10.1007/s12149-013-0777-6 24129541 PMC4328133

[11] Grueneisen J, Schaarschmidt BM, Heubner M, et al. Integrated PET/MRI for whole-body staging of patients with primary cervical cancer: Preliminary results. Eur J Nucl Med Mol Imaging. 2015; 42(12): 1814–24. DOI: 10.1007/s00259-015-3131-5 26199113

[12] Grueneisen J, Schaarschmidt BM, Heubner M, et al. Implementation of FAST-PET/MRI for whole-body staging of female patients with recurrent pelvic malignancies: A comparison to PET/CT. Eur J Radiol. 2015; 84(11): 2097–102. DOI: 10.1016/j.ejrad.2015.08.010 26321491

[13] Queiroz MA, Kubik-Huch RA, Hauser N, et al. PET/MRI and PET/CT in advanced gynaecological tumours: Initial experience and comparison. European Radiology. 2015; 25(8): 2222–30. DOI: 10.1007/s00330-015-3657-8 26017734

[14] Stecco A, Buemi F, Cassara A, et al. Comparison of retrospective PET and MRI-DWI (PET/MRI-DWI) image fusion with PET/CT and MRI-DWI in detection of cervical and endometrial cancer lymph node metastases. La Radiologia medica. 2016; 121(7): 537–45. DOI: 10.1007/s11547-016-0626-5 27033474

[15] Kirchner J, Sawicki LM, Suntharalingam S, et al. Whole-body staging of female patients with recurrent pelvic malignancies: Ultra-fast ^18^F-FDG PET/MRI compared to ^18^F-FDG PET/CT and CT. PloS one. 2017; 12(2): e0172553 DOI: 10.1371/journal.pone.0172553 28225831 PMC5321458

[16] Mongula JE, Bakers FCH, Voo S, et al. Positron emission tomography-magnetic resonance imaging (PET-MRI) for response assessment after radiation therapy of cervical carcinoma: a pilot study. EJNMMI Res. 2018; 8(1): 1 DOI: 10.1186/s13550-017-0352-6 29292485 PMC5748389

